# Azadiradione, a Component of Neem Oil, Behaves as a Superoxide Dismutase Mimic When Scavenging the Superoxide Radical, as Shown Using DFT and Hydrodynamic Voltammetry

**DOI:** 10.3390/biomedicines11113091

**Published:** 2023-11-18

**Authors:** Raiyan Sakib, Francesco Caruso, Stuart Belli, Miriam Rossi

**Affiliations:** Department of Chemistry, Vassar College, Poughkeepsie, NY 12604, USA

**Keywords:** neem oil, DFT, hydrodynamic voltammetry, superoxide dismutase, superoxide scavenging, terpenoids

## Abstract

The neem tree, *Azadirachta indica*, belongs to the Meliaceae family, and its use in the treatment of medical disorders from ancient times to the present in the traditional medical practices of Asia, Africa and the Middle East is well-documented. Neem oil, extracted from the seeds of the fruit, is widely used, with promising medicinal benefits. Azadiradione, a principal antioxidant component of the seeds of *A. indica*, is known to reduce oxidative stress and has anti-inflammatory effects. To directly measure the antioxidant ability of neem oil, we used Rotating Ring Disk Electrode (RRDE) hydrodynamic voltammetry to quantify how it can scavenge superoxide radical anions. The results of these experiments show that neem oil is approximately *26 times stronger* than other natural products, such as olive oil, propolis and black seed oil, which were previously measured using this method. Next, computational Density Functional Theory (DFT) methods were used to arrive at a mechanism for the scavenging of superoxide radical anions with azadiradione. Our work indicates that azadiradione is an effective antioxidant and, according to our DFT study, its scavenging of the superoxide radical anion occurs through a reaction mechanism in which azadiradione mimics the antioxidant action of superoxide dismutase (SOD). In this mechanism, analogous to the SOD enzymatic reaction, azadiradione is regenerated, along with the production of two products: hydrogen peroxide and molecular oxygen. This antioxidant process provides an explanation for azadiradione’s more general and protective biochemical effects.

## 1. Introduction

The neem tree, *Azadirachta indica*, belongs to the Meliaceae family of plants, which are found in tropical and subtropical regions of the world. Its use in the treatment of medical disorders from ancient times to the present is well-documented. In fact, extremely ancient medicinal practices from the Indian subcontinent, such as Indian Siddha medicine, highlight the importance of the neem tree as a medicinal plant [1,2]. Evidence for the long history of neem use can be seen in the findings of neem leaves among the archaeological treasures of the Harappan Indus Civilization, uncovered in the 1921 explorations [2]. Today, the use of neem products to treat skin diseases including chicken pox, caused by varicella zoster virus, is still common. Until its eradication, neem was used in the treatment of smallpox (variola virus) [2]. Today, neem products are widely used in the traditional medical practices of Asia, Africa and the Middle East [3], and a number of more recent reviews describing the extensive biomedical properties of the neem tree are available [4,5,6,7,8]. To date, research in pharmaceutical and medicinal chemistry has explored the molecular components of the neem tree and its products in an effort to derive chemical mechanisms for its exceptional therapeutic properties. Different constituents of the tree have proved to be rich sources of natural compounds, with neem leaves, seeds and oil extensively used for biomedical purposes [9,10,11].

The neem tree is a rich source of plant secondary metabolites, including many terpenes that have promising biological activities, as in the biosynthesis of important compounds such as steroids and sterols [12,13,14]. Limonoids are a sub-group of triterpenes found mainly in the Meliaceae and Rutaceae plant families; their name arose due to the fact that they were first identified from the *Citrus* family (Rutaceae). An important review describing the chemistry and biological properties of the large number of Meliaceae limonoids was published in 2011 [15]. Of interest, the neem tree serves as the source of several limonoids with significant biological activities, including insect antifeeding action. For example, azadirachtin has been identified as the principal active insecticidal species [16] against a broad range of insects while also being non-toxic towards mammals, thus giving a scientific basis for the wide popularity of neem tree products as insect repellants. Gedunin is a potent larvicide, antifungal and antitumor agent [15,17], while nimbolide has shown antimalarial and antitumor properties [13,18,19,20]. Nimbolide has also been reported to have a positive effect in animal studies in treating gestational diabetes by boosting total antioxidant capacity and antioxidant enzymes, such as superoxide dismutase (SOD), glutathione peroxidase (GPx), glutathione S-transferase (GST) and catalase (CAT) [21]. Gedunin was described as a robust and non-toxic Nrf2 activator acting against oxidative stress, since Nrf2 is a key transcriptional regulator of antioxidant defense and detoxification [22]. The ROS scavenging of gedunin and its ability to reduce oxidative stress to augment current type 2 diabetes therapies were described [23]. Gedunin was also the first identified antimalarial limonoid agent from the neem tree [24]. In summary, the literature comprises many reports on the neem limonoids azadirachtin, gedunin, and nimbolide.

The impressive biomedical consequences of using neem tree parts are often accompanied by descriptions of antioxidant and anti-inflammatory activities [2,25,26,27,28]. For example, *A. indica* (neem) oil significantly reduced oxidative stress and lipid peroxidation, leading to mice liver damage when exposed to the mycotoxin Ochratoxin A [29]. The antioxidant effects of neem leaf fractions against H_2_O_2_-induced lipid peroxidation and DNA damage were attributed to their ability to inhibit various free radicals [2,30]. 

**Figure 1:**
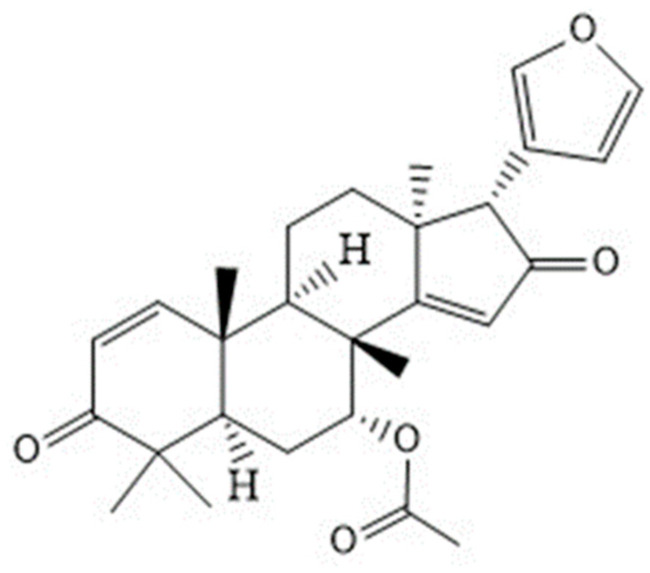
Azadiradione molecular structure.

The composition/concentration of neem tree limonoids was shown to vary according to different factors including ripening [31]. Another study used HPLC and NMR data on neem components in five different stages of ripening to quantify, isolate and identify the limonoids [32]. This report showed that the azadirachtin content was very low in green berries (3%), and it increased to 13% on ripening. The compound having the highest concentration (59%) in green berries, which gradually decreased to 3% on fruit ripening, was azadiradione, as shown in Figure 1, a major but less-studied limonoid constituent.

This compound, azadiradione, also has important biological actions such as reducing oxidative stress and anti-inflammatory effects [8,28,33,34]. In neurodegenerative diseases such as polyglutamine-based diseases and Alzheimer’s, Huntington’s and Parkinson’s diseases, the current treatment options are limited. Azadiradione is of therapeutic interest because it is an inducer of Heat Shock Factor 1 activity in cell and Drosophila models [35]. A study performed on the deacetyl derivative of azadiradione showed that this molecule exerted a protective effect on zebrafish larvae that were stressed through exposure to lipopolysaccharides, LPS (the increased circulation of LPS leads to the induction of oxidative stress and a systemic inflammatory response). This was accomplished through its scavenging of the free radicals produced during oxidative stress, thereby reducing the inflammatory response [36].

Indeed, azadiradione has a triterpenoid structure similar to that of another biologically active antioxidant plant species previously studied by us, celastrol [37]. Consequently, our objective in this work was to study the antioxidant properties of azadiradione and the commonly available neem oil in which it is found, so that we could obtain general insights into the chemistry and biological activities of this meliaceous limonoid. To this end, we measured the superoxide radical scavenging ability of neem oil directly using Rotating Ring Disk Electrode (RRDE) hydrodynamic voltammetry. To arrive at a reaction mechanism through which azadiradione can effect this scavenging of the superoxide radical anion, we used DFT computational methods. 

## 2. Materials and Methods

### 2.1. Hydrodynamic Voltammetry (RRDE)

**Figure 2:**
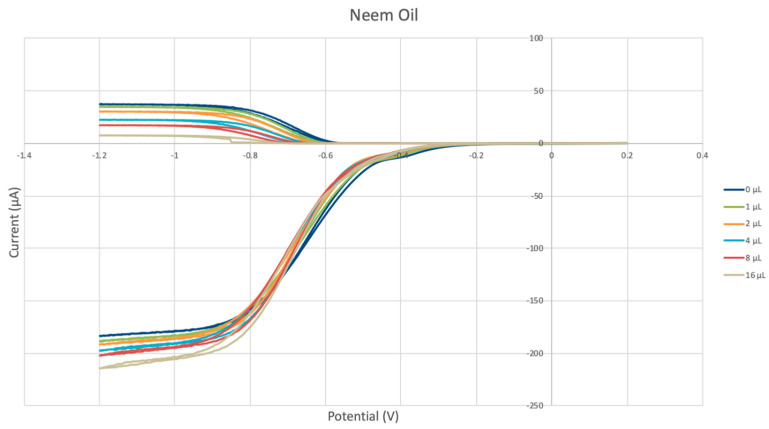
RRDE data for neem oil. The bottom part (negative current) shows the formation of superoxide detected at the disk electrode; the top (positive current) shows that the superoxide detected at the ring electrode decreases after adding neem oil aliquots.

A commercial sample of 100% cold-pressed neem oil was used as received (Milania, Amazon.com). For the experiment, the electrochemical cell containing a solution of 0.1 M dried tetrabutylammonium bromide, TBAB (Sigma-Aldrich, St. Louis, MO, USA), dissolved in 50 mL dimethyl sulfoxide, DMSO, anhydrous, ≥99.9% (Sigma-Aldrich, St. Louis, MO, USA), was bubbled with dry O_2_/N_2_ (35%/65%) for five minutes to establish the required dissolved molecular oxygen level. The rotation setting used for the rotation of the Au/Au disk electrode was fixed at 1000 rpm, and the potential sweep was applied to the disk from 0.2 V to −1.2 V and then reversed to 0.2 V, while the potential of the ring electrode was held invariable at 0.0 V. The disk voltage sweep rate was positioned at 25 mV/s. The molecular oxygen reduction peak (Reaction 1) was detected around −0.6 V at the disk electrode. Meanwhile, the oxidation (Reaction 2) occurred at the ring electrode. An initial blank solution consisting of bubbled O_2_, the electrolyte TBMB and DMSO alone (in the absence of neem oil), was run, and the ratio of the ring/disk current was defined as the “efficiency”. Next, a neem oil antioxidant aliquot was provided, as indicated in Figure 2. The solution in the voltaic cell was bubbled with the gas mixture for 5 min, an updated voltammogram was recorded, and the corresponding efficiency was obtained. In this way, the rate at which the increasing concentrations of the antioxidant sequester the generated superoxide radicals during the electrochemical reaction is determined as each additional antioxidant aliquot is added. Aftermath software Release 1.6.10523 was used to record the results from each run, represented as voltammograms showing the current vs. potential graphs. These were later analyzed using Microsoft Excel. The volume amount used in each of the aliquots is indicated in the related RRDE graph. Finally, the decreasing slope of the curve, describing the overall decrease in efficiency with the incremental addition of the antioxidant, serves as a quantitative measure of the antioxidant activity of neem oil. Any decrease in the collection efficiency is anticipated to be due to the amount of superoxide consumed by the neem oil. This method was developed in our laboratory [38]. In our RRDE voltammetry experiment, the generation of the superoxide radical anions occurred through a reduction at the disk electrode, while the reverse oxidation reaction of the residual superoxide radicals (those that remain unreacted) were detected at the ring electrode.

Reaction 1: Reduction of molecular oxygen occurring at the disk electrode

O_2_ + e^−^ → O_2_^•−^ (1)

Reverse Reaction 2: Oxidation of superoxide radicals at the ring electrode

O_2_^•−^ → O_2_ + e^−^ (2)

### 2.2. Computational Study

Calculations were run using Dmol^3^, a program in the Biovia package (Dassault Systèmes, San Diego, CA, USA). This program utilizes Density Functional Theory (DFT) to calculate energy, geometry, and frequencies, implemented in Materials Studio 7.0 [39]. We employed the double numerical polarized (DNP) basis set including all the occupied atomic orbitals plus a second set of valence atomic orbitals, as well as polarized d-valence orbitals [40]. Correlation generalized gradient approximation (GGA) was used, including BLYP correlation and Becke exchange [41]. All electrons were treated explicitly, and the real space cutoff of 5 Å was set for the numerical integration of the Hamiltonian matrix elements. The self-consistent field convergence criterion was established for the root mean square variation in the electronic density to be less than 10^−6^ electron/Å^3^. No solvent effects were included in these calculations. The convergence criteria applied during geometry optimization were 2.72 × 10^−4^ eV for energy and 0.054 eV/Å for force.

## 3. Results and Discussion

### 3.1. Electrochemical Study

**Figure 3:**
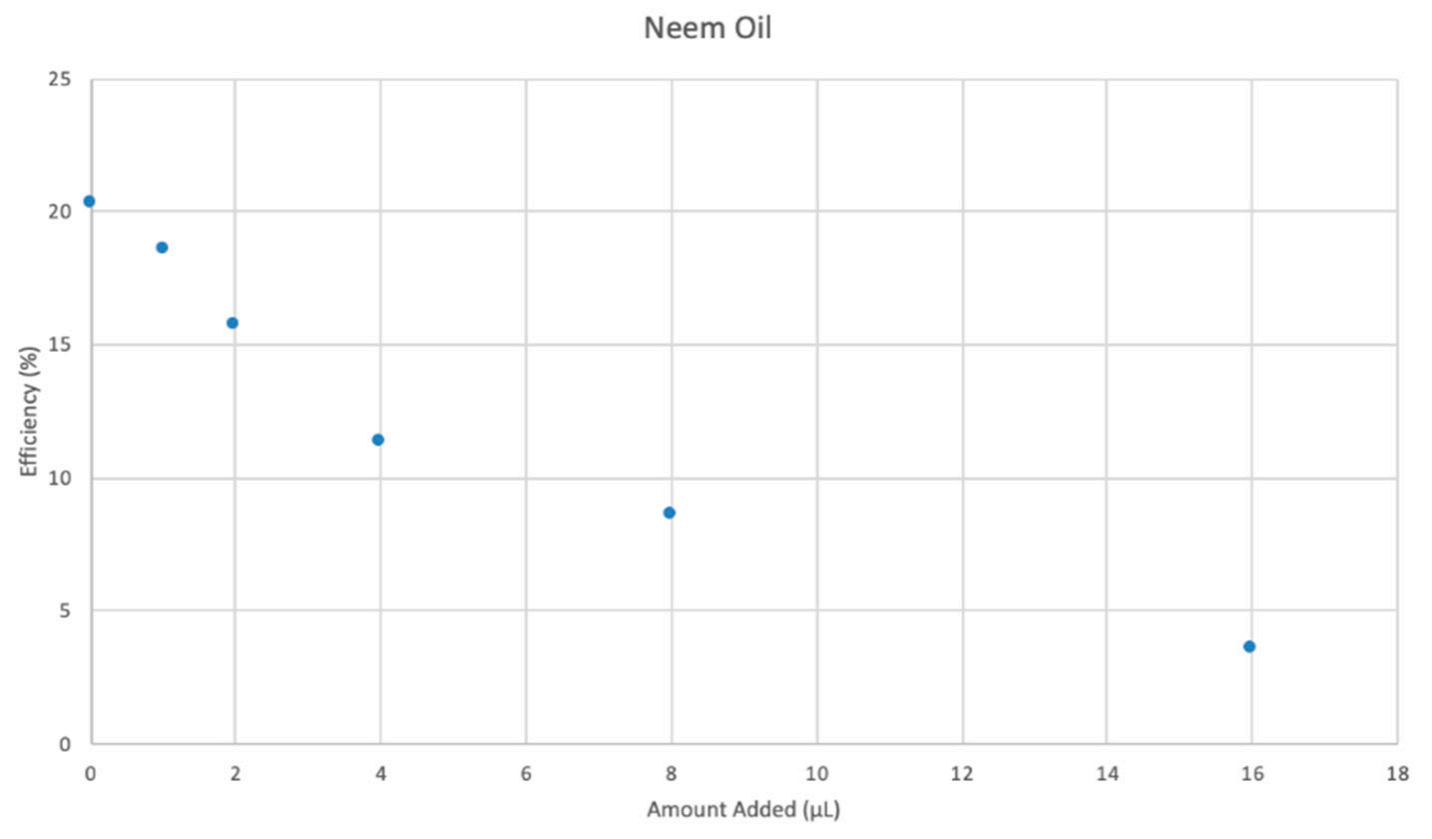
Collection efficiency of RRDE neem oil. The ring current/disk current (% Efficiency) at each concentration vs. added amount of neem oil shows a decreasing trend.

The Rotating Ring Disk Electrode (RRDE) method is an electroanalytic technique related to standard cyclic voltammetry. It is effective in rapidly detecting stable species during electrochemical reactions and has the advantages of a high sensitivity and low cost. The antioxidant capability of neem oil for the superoxide radical was studied using the RRDE method. Essentially, the superoxide radical is generated in a voltaic cell using anhydrous DMSO as a solvent, together with an electrolyte, and by bubbling a controlled amount of oxygen gas, allowing for solution saturation. The superoxide radical is obtained at a sufficiently negative potential so that the O_2_ can capture an electron from the working disk electrode to form the anionic superoxide radical, O_2_ + e^−^ → O_2_^•−^, as in Reaction (1). From the RRDE graph in Figure 2, it can be seen that increasing amounts of neem oil decrease and even almost deplete the superoxide concentration in the voltaic cell. That is, the signal detected at the ring electrode shows that the superoxide is still existing and not consumed by the antioxidant. This is located at the upper part of the graph, and we see that upon adding 16 μL of neem oil, the superoxide concentration is almost completely eliminated. The collection efficiency graph also shows this effect in Figure 3.

**Figure 4:**
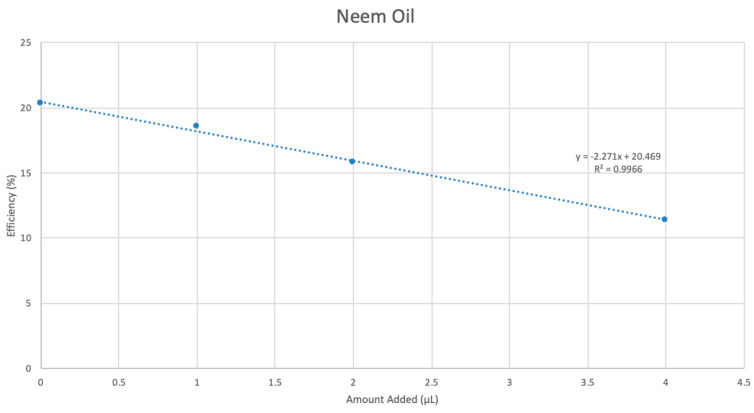
Collection efficiency of neem oil, limited to the first four data points, shows the linear trend y = −2.271x + 20.469, R^2^ = 0.9966.

The clear-cut parameter defining antioxidant superoxide scavenging ability is the slope of the collection efficiency graph, which is shown in Figure 4. A comparison of the slopes of neem oil and other previously studied natural products is shown in Table 1. We highlight that the steeper the slope is, the stronger the antioxidant capacity of the studied scavenger species will be. 

The previously examined oils have slopes of approximately −0.08. Neem oil has a slope approximately *26 times stronger*, −2.271. Among these studied oils, extra virgin olive oil is well-known to have a low concentration of polyphenols, from 50 to 1000 mg/kg [45], since it mostly contains fatty acids and esters, which cannot provide an antioxidant action. Therefore, the limited amount of polyphenols in olive oil are not able to scavenge as much superoxide as those antioxidants in neem oil. It can be concluded that the effective components of scavenging in neem oil are in higher concentration than those in olive oil, black seed oil and propolis. Additionally, some neem oil components are more effective scavengers than the tyrosol and hydroxytyrosol found in extra virgin olive oil. Section 3.2 explores these details further.

### 3.2. DFT Study

**Figure 5:**
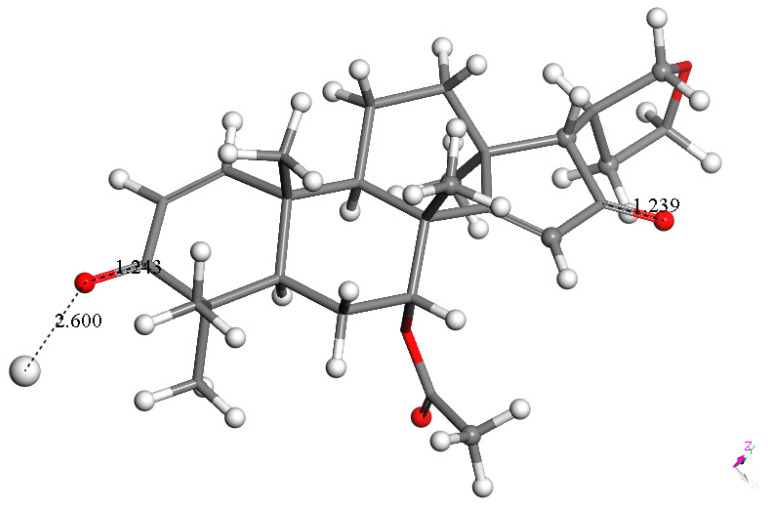
Azadiradione was DFT-minimized, and a proton was placed through van der separation forces using the O(carbonyl) associated with the cyclohexene ring, 2.60 Å.

**Figure 6:**
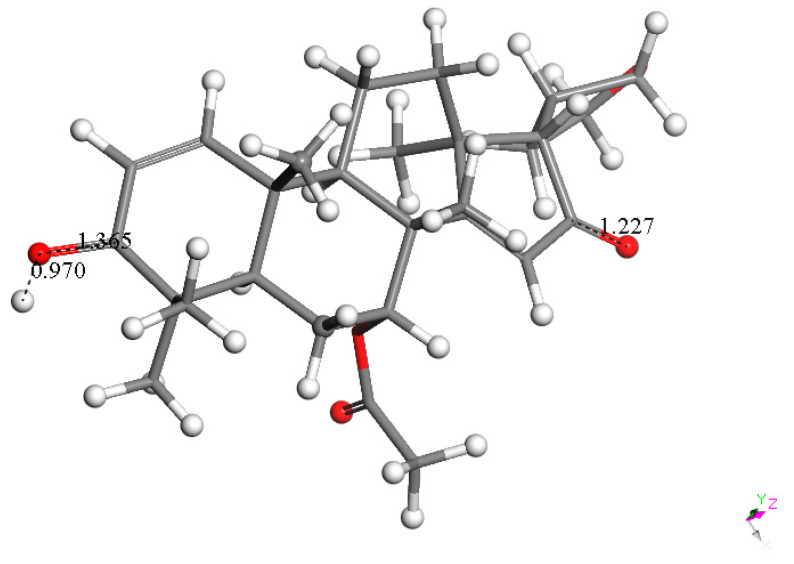
Upon DFT minimization of the azadiradione, as shown in Figure 5, the O(carbonyl) captures the added proton without any energy barrier, with an O-H bond distance of 0.970 Å, while the associated C-O bond becomes longer, at 1.365 Å, compared to the other C=O carbonyl, 1.227 Å.

**Figure 7:**
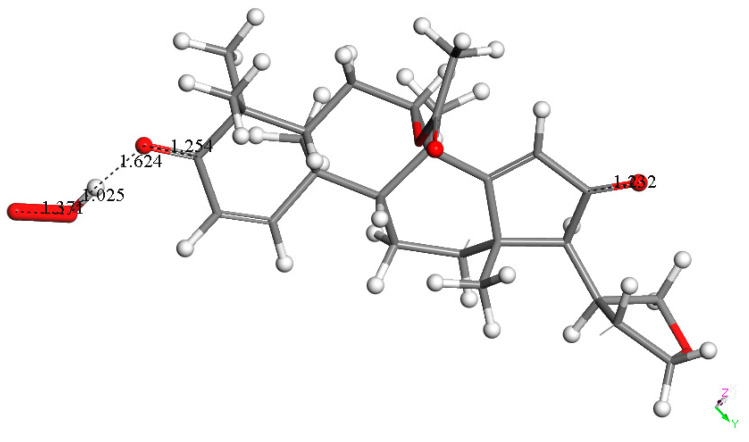
Near the proton added to azadiradione in Figure 6, a superoxide radical (O-O bond distance 1.373 Å) is initially placed through van der Waals forces, 2.60 Å apart (not shown). Upon DFT minimization, the proton becomes linked to superoxide, forming a HO_2_ species, which results in its detachment from the remaining azadiradione neutral radical, O---H distance = 1.624 Å.

**Figure 8:**
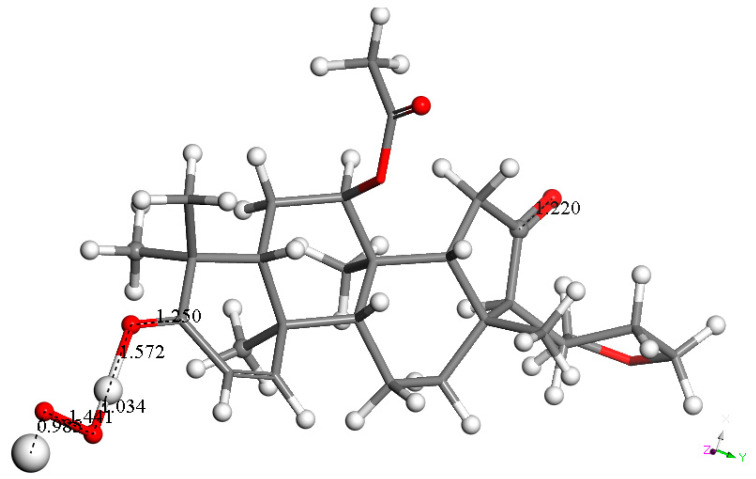
A second proton is initially placed near the more exposed oxygen atom in HO_2_, 2.60 Å (not shown), making the whole arrangement a 1+ charged radical system (from 2 protons plus the reacted negative superoxide). Upon DFT optimization, H_2_O_2_ forms and detaches from the organic moiety, 1.572 Å, with the adjacent carbonyl having an only slightly longer CO bond length, 1.250 Å, than the carbonyl at the opposite end of the molecule, 1.220 Å.

The molecular structure of azadiradione was DFT-energy-minimized using starting coordinates from its crystal structure [46]. After a proton was placed at the van der Waals separation from the six-membered ring of azadiradione O(carbonyl), 2.60 Å, Figure 5, the whole arrangement was DFT minimized, and the proton resulted bound to the oxygen, 0.970 Å, Figure 6. Next, a superoxide was placed using van der Waals separation through the added proton in Figure 6, and DFT minimization showed the formation of a HO_2_ moiety, separated from the remaining azadiradione neutral radical by 1.624 Å, as shown in Figure 7. Next, the more exposed oxygen of HO_2_ was positioned 2.60 Å from an additional proton, making the whole radical system charged (+1), from two protons plus the reacted superoxide. Upon DFT optimization, H_2_O_2_ formed and detached from the organic moiety, 1.572 Å, showing the neighboring carbonyl (C-O bond of 1.254 Å), which was very similar to the carbonyl at the opposite end, as in Figure 8. 

**Figure 9:**
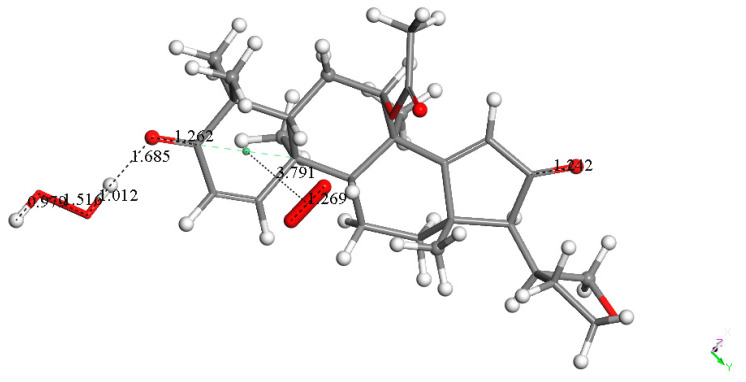
To the arrangement shown in Figure 8, a 2nd superoxide radical was π-π-posed at the van der Waals separation, 3.50 Å from the center of the ring, making the system neutral and non-radical (not shown). After DFT optimization, the superoxide donated its unpaired electron to the ring, forming a molecule of O_2_ (O-O bond 1.269 Å, much shorter than the 1.373 Å distance in the superoxide) that then detached from azadiradione, 3.791 Å. Meanwhile, the separation distance of H_2_O_2_ from azadiradione increased, 1.685 Å. Thus, after the reaction of azadiradione with two superoxide radicals (Figure 7 and Figure 9) and two protons (Figure 6 and Figure 8), azadiradione is reformed and becomes ready for an additional cycle of superoxide radical scavenging. The reaction products are H_2_O_2_ (Figure 7) and O_2_ (Figure 9). Scheme 1 displays the whole process.

**Scheme 1:**
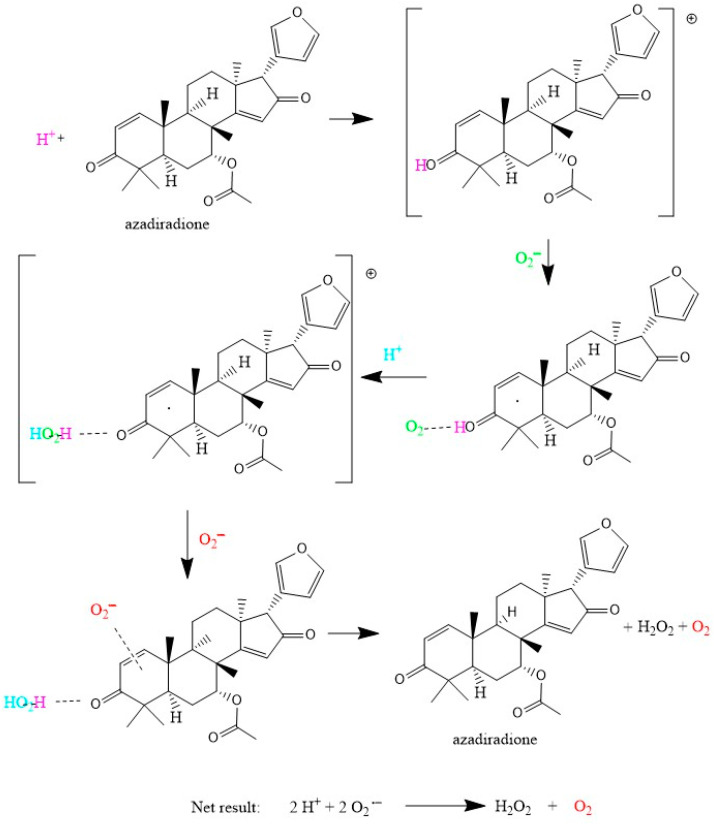
Azadiradione scavenging of superoxide follows the same pattern as the superoxide dismutase enzyme, Reaction (3). No energy barriers were observed for all reactions. In the first step, the initially van-der-Waals-separated proton and O(carbonyl) are established, and upon DFT geometry optimization, the corresponding ΔG_reaction_ is −552.3 kcal/mol (top). Next, a green-colored superoxide is placed (van-der-Waals-separated to join the previously added proton), and C=O-H-O_2_ is established (ΔG = −1362.5 kcal/mol) (**center right**). To the center right species, a second (turquoise-colored) proton is placed by the most exposed O atom in the O_2_-H-O-C moiety, and applying DFT minimization, the H_2_O_2_ moiety forms and slightly detaches from O(carbonyl), ΔG = −117.3 kcal/mol (**center left**). Next, a second (pink-colored) superoxide is π-π-attached to the cyclohexene ring (**bottom left**), and upon DFT minimization, the superoxide transfers its unpaired electron to the ring, and O_2_ is eliminated along with the previously formed H_2_O_2_, ΔG = −17.3 kcal/mol. Thus, the final products are indicated, including reformed azadiradione, ready to start another catalytic cycle (**bottom right**).

Next, in the arrangement shown in Figure 8, another superoxide was placed at the van der Waals separation, 3.50 Å above the ring containing the double bond, to explore a π-π interaction. Upon DFT minimization, the original distance between the O atoms in the attacking superoxide, 1.373 Å, was shortened to 1.269 Å, which is approximately the bond distance for a molecule of O_2_, as shown in Figure 9. Meanwhile, this molecule of O_2_ was rejected from the system at 3.791 Å, and H_2_O_2_, already formed in Figure 8 (separated 1.572 Å), further detached from the organic moiety at 1.685 Å. It can be concluded that the unpaired electron of the second superoxide was directed towards the double bond in the cyclohexene ring. The consequence of this process was the consumption of two superoxide radicals and two protons to obtain O_2_, H_2_O_2_ and reformed azadiradione, which was ready for the additional scavenging of superoxide. The related Reaction (3) is the same as that of superoxide dismutase and fully described in Scheme 1, which also includes the involved ΔG_reaction_ for each step. For instance, the capture of the proton by the azadiradione O(carbonyl) has ΔG_reaction_ of −552.3 kcal/mol (Figure 6), and for the oxidation of the superoxide (Figure 9), ΔG_reaction_ is −17.3 kcal/mol. No energy barriers were observed for all the reactions.

2 O_2_^•−^ + 2H^+^ → O_2_ + H_2_O_2_ (3)

We can conclude that by incorporating a proton, the azadiradione carbonyl, the C=O bond distance of which is 1.243 Å, lengthens to 1.365 Å, which is a typical single C-O bond length, and so a C-O-H moiety is established (Figure 6). Next, a superoxide anion interacts with the previously added proton in the reacting moiety, and later, a second proton further reacts to form H_2_O_2_, which restores the C=O bond in azadiradione, 1.250 Å (Figure 8). However, the charge of the whole molecular system is +1 (from two protons and −1 from the superoxide), and, more importantly, the system also has an uneven number of electrons (it is a radical) due to the reacting superoxide radical. It is therefore not surprising that the reaction of a second superoxide anion is feasible, making the whole system non-radical (the new product has an even number of electrons) and neutral. The interesting redox property of this second superoxide interaction is the eventual elimination of a molecule of O_2_, as seen in the energy minimized ensemble resulting in a π-π separation of 3.791 Å to the cyclohexene centroid (Figure 9), longer than the original van der Waals separation of 3.5 Å, while more importantly, the superoxide shortens its O-O bond to 1.269 Å, which is consistent with a molecule of O_2_, that is, it is shorter than the O-O bond distance of the reacting superoxide, 1.373 Å. Thus, interestingly, the second superoxide is not an oxidizing agent; rather, it oxidizes itself. The receptor of this extra electron seems to be the double bond in the cyclohexene moiety. As will be explained later, in a previous study, we described a similar reducing superoxide action through a π-π interaction with the pyrone ring of isoflavones. In summary, the final result is a dismutase action, e.g., the first superoxide incorporates two protons to form H_2_O_2_ (a typical reaction for antioxidants found in fruit and vegetables as for instance flavonoids), while the second superoxide is a reducing reagent, as it becomes oxidized on releasing its unpaired electron.

**Figure 10:**
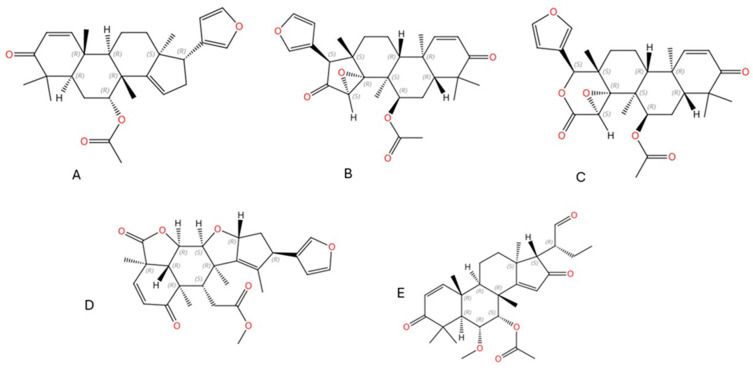
Molecular structures of tetracyclic terpenoid neem oil compounds closely related to azadiradione, e.g., containing the cyclohexene-carbonyl moiety: azadirone (**A**), epoxyazadiradione (**B**), gedunin (**C**), nimbolide (**D**) and zafaral (**E**).

Other tetracyclic terpenoids found in neem oil may also contribute to superoxide scavenging, among them azadirone, epoxyazadiradione, gedunin, nimbolide and zafaral [6], shown in Figure 10, which all contain the cyclohexene carbonyl moiety responsible for antioxidant activity in azadiradione. In fact, this moiety is conserved after the deacetylation of the epoxy group coupled with the substitution of a hydroxy group, with the resulting molecule showing enhanced free radical scavenging and experimental antioxidant activities [36], in agreement with our DFT calculations, as shown in Scheme 1. 

The antioxidant properties of neem oil compounds have been explored in the literature. For instance, nimbolide exhibited a concentration-dependent anti-radical scavenging activity and is a potent antioxidative agent [17]. Moreover, nimbolide decreased oxidative damage by reducing ROS accumulation in cells while increasing the activity of radical scavenging enzymes such as superoxide dismutase (SOD), glutathione peroxidase (GPx), glutathione S-transferase (GST) and catalase (CAT) [47]. The antioxidant action of nimbolide was also recently substantiated through its ability to increase the antioxidant enzyme activity of SOD, GST and CAT in a rat model of gestational diabetes [21]. In contrast, the pro-oxidant effects of nimbolide, with its causation of oxidative stress through the suppression of antioxidant enzyme activity, appears to be beneficial and part of its cytotoxic action [20]. Additionally, there is another report of nimbolide-induced oxidative stress with a significant decrease in antioxidant enzyme activity in rat spermatozoa [48]. The nimbolide mechanism of action, with respect to ROS, thus remains unclear.

## 4. Conclusions

The results of our data, taken together, indicate that azadiradione, a component of neem fruit, is an effective antioxidant, and a mechanism for its scavenging of the superoxide radical anion has been proposed. 

In this study, we have shown that azadiradione is a strong antioxidant, along with neem oil. Additionally, these electrochemistry experiments showed that neem oil is approximately *26 times stronger* than other natural products, such as extra virgin olive oil [42], propolis [44] and black seed oil [43], which were previously measured using this method. Moreover, according to our DFT study, azadiradione mimics the antioxidant action of superoxide dismutase (SOD), suggesting an explanation for its more general and protective effects as an antiplasmodial, anti-insecticidal, anti-inflammatory, antifungal and antitumor product in aging-related conditions as Parkinson’s disease [33]. SOD are a family of metalloproteins that protect cells against oxidative stress by catalyzing the chemical reaction for the dismutation of the superoxide radical anion (O_2_^●−^) into molecular oxygen and hydrogen peroxide through metal ion redox chemistry, as described in Reaction (3) [49]. In the literature, the active site interaction of two antioxidant enzymes, superoxide dismutase and xanthine oxidase, with three small molecules found in *A. indica* extract, protocatechuic acid (−)− epicatechin and gallic acid, has been described [50]. 

We also observed a different correlation: the neem oil component azadiradione can mimic SOD activity *directly* by interacting with the superoxide radical anion to produce hydrogen peroxide and the oxygen molecule. Because SOD provides a defense against oxidative stress under physiological and disease conditions, the finding of natural products that can mimic SOD activity is of interest for their therapeutic potential. Thus far, most SOD mimics have been metal complexes [51], and the reactions depend on redox active metals, whereas azadiradione and other plant-derived compounds can achieve this effect through initial intermolecular interactions such as hydrogen bonding and stacking interactions.

Here, these SOD mimics carry out the dismutation of superoxide in this manner: overall, there are two superoxide anions reacting, the first capturing two H atoms and forming H_2_O_2_, while the second superoxide releases its unpaired electron to the antioxidant. 

The results of this work add to our earlier studies, which demonstrated that some organic natural products such as isoflavones [52], galangin [44] and other natural products [53] can effectively act as SOD mimics. Our results suggest the possibility that azadiradione can mimic SOD action, thus facilitating an explanation for its ability to assist in lowering superoxide concentrations. Because oxidative stress is associated with a variety of disease states, we plan to conduct future research aimed at illuminating this important biomedical activity using non-toxic natural compounds. 

Our results confirm that the RRDE cyclic voltammetry experiment has several advantages over traditional methods for measuring antioxidant activity (e.g., the DPPH assay, which uses an unsuitable non-biological radical). Other methods used to generate the superoxide radical consist of measuring a product after superoxide consumption, in which adding an antioxidant will decrease the concentration of such a product, e.g., the superoxide concentration is measured indirectly. One of these methods is an enzymatic reaction using xanthine dehydrogenase [54], while a non-enzymatic option utilizes phenazine methosulphate, NADH and molecular oxygen [55]. In contrast, the RRDE can be used to directly measure antioxidant superoxide scavenging ability in the sample cell after superoxide production *in situ*. 

## Figures and Tables

**Table 1:** Comparison of slopes of the plant products studied in this work and other natural products analyzed with the RRDE method.

Olive Oil [42]	Black Seed Oil [43]	Propolis [44]	Neem Oil
−0.0838	−0.078	−0.0864	−2.271
